# Cross-Cultural Validation of the High Blood Pressure Health Literacy Scale in a Chinese Community

**DOI:** 10.1371/journal.pone.0152182

**Published:** 2016-04-26

**Authors:** Qinghua Zhang, Feifei Huang, Zaoling Liu, Na Zhang, Tanmay Mahapatra, Weiming Tang, Yang Lei, Yali Dai, Songyuan Tang, Jingping Zhang

**Affiliations:** 1 Xiang Ya Nursing School, Central South University, Changsha, Hunan, China; 2 School of Nursing, XinJiang Medical University, Urumqi, XinJiang, China; 3 School of Nursing, FuJian Medical University, Fu Zhou, Fu Jian, China; 4 School of Public Health, XinJiang Medical University, Urumqi, XinJiang, China; 5 School of Public Health, University of Hong Kong, Hong Kong, China; 6 Department of Epidemiology, Fielding School of Public Health, University of California, Los Angeles, United States of America; 7 University of North Carolina Project-China, Guangzhou, China; The University of Tokyo, JAPAN

## Abstract

**Background:**

Considering the importance of health literacy (HL) for the maximum yield from the hypertension control programs, development of a reliable and valid instrument of hypertension-related HL is critical. This study aimed to translate and validate the High Blood Pressure-Health Literacy Scale (HBP-HLS) into Chinese (C-HBP-HLS) and evaluate its psychometric properties in Chinese context.

**Method:**

Between June 2013 and January 2014, a cross-sectional study was conducted among recruited hypertensive patients belonging to the Han and Kazakh-Chinese communities in Urumqi, Xinjiang, China.

**Results:**

A pilot sample (n = 242) was selected for the exploratory factor analysis of the translated and modified instrument. Another sample (n = 308) was recruited for the confirmatory factor analysis. C-HBP-HLS consisted of five dimensions (Print Health Literacy, Medication Label, Understanding Ability, Newest Vital Sign Test, and Avoiding Food Allergy) containing 15 items, accounting for 77.7% of the total variance. The 5-factor model demonstrated a good overall fit. The scale-level content validity index was 0.85. Cronbach’s alpha of the overall scale was 0.78 and test-retest reliability was 0.96. Education level had a strong positive correlation with the scores for items Q1, Q2, and Q3(r = 0.481, 0.492, 0.475, respectively). Health Literacy scores among Kazakh patients were significantly lower than Han (7.13±7.90 vs. 30.10±13.42, Z = -14.573, *P*<0.001).

**Conclusion:**

C-HBP-HLS demonstrated suitable factor structure and robust psychometric properties for measuring health literacy level among hypertensive patients in China.

## Introduction

Owing to its contributory role in the global burden of diseases, hypertension is a major public health concern worldwide and China is no exception. The Chinese national survey report indicates that the prevalence of hypertension has increased significantly from 14.5% in 1991 to 29.6% in 2010 [1, 2], higher than the global prevalence (27%)[3], resulting in a direct medical cost of approximately US $ 3.37 billion [20.2 billion Ren Min Bi (RMB)] attributable to hypertension [4]. In the far west of China, in Xinjiang province, the proportion of ethnic minorities is about 60% (12.98 million out of 21.81 million total population). Here the prevalence of hypertension is as high as 44.2% [5], significantly higher than the national estimate (29.6%) for China [1]. Across ethnicities, the prevalence is highest among Kazakhs (54.6%), followed by Hans (36%) [5]. Based on these estimates, it is obvious that hypertension has become a serious public health problem, particularly among Kazakh ethnic minority population and Han ethnicity in western China. Thus evaluation of newer interventions to determine their effectiveness appears to be critical for efficient policy-making targeting hypertension control.

Referred as the sixth vital sign [6], health literacy (HL) has received increasing attention in the healthcare field recently. The term of HL refers to the degree to which individuals have the capacity to obtain, process, and understand basic health information and services necessary to take appropriate health decisions [7]. Previous studies emphasized that low HL was ubiquitous among ethnic minorities [8, 9] and was positively associated with the self-management and quality of life of the patients [10]. Patients having low HL were generally 1.5 to 3 times more likely to experience poor health outcomes as opposed to those with better HL [11]. Low HL is known to have negative influences on the ability of searching health information, understanding medical instructions, adopting healthy behavior and managing one’s own health, cumulatively contributing to unnecessary hospital costs among patients of non-communicable diseases [12]. HL has also been observed to be a stronger predictor of poor health outcomes compared to other socio-demographic factors like age, income, employment status, education, and race [9]. Individuals with adequate HL can utilize the available health resources effectively, and can manage their own health scientifically [10]. Thus keeping all these factors in mind, it is not surprising to note that in recent years, HL is getting progressively increasing attention as a significant independent predictor for the effectiveness of the blood pressure control programs [13]. Consequently, assessment of the HL level of the hypertension patients at the time of initial contact with the health system became important.

Considering the importance of HL for the maximum yield from the hypertension control programs, development of a reliable and valid instrument of hypertension-related HL is critical. This instrument is not only helpful to determine the relevant HL level accurately, but also can help us to develop appropriate educational intervention to improve the HL as well as resultant self-management for efficient control of hypertension. For example, appropriateness of the language and health education materials can easily be customized according to individual HL level to meet the needs of different patients. Currently, a number of instruments are available for measuring HL, such as the Test of Functional HL in Adults (TOFHLA) [14], the Rapid Estimate of Adult Literacy in Medicine(REALM) [15], HL in Dentistry (HeLD) [16], and the High Blood Pressure-HL Scale (HBP-HLS) [17], etc.

The HBP-HLS is the only available hypertension-specific HL instrument, which has been developed based on the focus group studies and literature reviews. It has been validated among the Korean Americans [17] and is considered to be a reliable and valid tool for measuring and evaluating HL in the context of hypertension management [18, 19]. It consists of 43 items, including two domains: Print HL (30 items) and Functional HL (13 items). Print HL consists of 30 hypertension-related words arranged into three columns of increasing complexity. Participants are supposed to get 1 point for the correct pronunciations of each of these words. To assess functional HL, comprehension and decision-making in health-related issues in daily life are assessed after providing relevant information [17].

Tools in local language to measure hypertension-specific HL were unavailable for Chinese population. Instead the available Chinese HL measurement tools were used to address HL in general, for instance, HL questionnaire-66 items [20], and the Chinese HL Scale for Chronic Care (CHLCC) [21]. Thus owing to the lack of reliable and valid HL measuring tool, their remained a paucity of information regarding the HL among patients with hypertension, in China. Therefore, in order to address the increasing trend of hypertension and to develop a “HL-focused hypertension management program”, in this study, HBP-HLS was modified, translated from English to Chinese (C-HBP-HLS), and its initial psychometric properties were validated among Kazakh and Han population in XinJiang Province of China. The findings were expected to provide important insight for the healthcare providers, regarding the required strategies to enhance the effectiveness of hypertension-reduction intervention, improve the quality of life and prolong lifespan.

## Methods

### Study design

A cross-sectional descriptive survey was conducted along with cross-validation of exploratory factor analysis (EFA) and confirmatory factor analysis (CFA) to explore the appropriate construct of HBP-HLS in Chinese cultural context.

### Recruitment

A randomized cluster sampling strategy was used to recruit participants having hypertension from Han and Chinese-Kazakh communities in Urumqi, XinJiang, China, between June 2013 and January 2014. Subjects aged 18 years or more, having systolic blood pressure of 140mmHg or higher and/or diastolic blood pressure of 90mmHg or higher or taking antihypertensive medication, who had no cognitive disorders, and were willing to participate in the study and signed the written informed consent were considered eligible. Patients were excluded if they had dysgnosia and/or psychosis, or severe communication impairment.

In the current study, Print HL was assessed by hypertension-related words arranged in 3 groups and each group assumed as a separate item (hence altogether 3 items in Print HL), while functional HL assessment remained identical with the method used in HBP-HLS by Kim et al. Thus overall the modified HBP-HLS in Chinese (C-HBP-HLS) in this study consisted of 16 items and two domains.

It is widely acknowledged that at least 100 samples are required, in order to establish an accurate inference in EFA [22]. In addition, in order to evaluate CFA, a minimum sample size of 200 is needed to gain reliable results[23]. According to the inclusion and exclusion criteria, a sample of (pilot sample) 242 participants were recruited from 270 eligible patients (response rate of 89.6%) for the exploratory study. In addition, 37 hypertension patients were selected for evaluation of the test-retest reliability, with a response rate of 100%. Another sample (validation sample) of 308 participants was recruited from 350 eligible participants (response rate of 88.0%) to confirm the findings from the exploratory study.

### Ethical considerations

This research was approved by the Ethics Committee of the First Affiliated Hospital of Xinjiang Medical University (Letter Number:20130216–134), and it was conducted according to the standards of the Declaration of Helsinki.

### Procedure and data collection

After obtaining the authorization from the original author from Nursing School of Johns Hopkins University, and the informed consent from each participant, four trained bilingual research assistants (RAs) conducted face to face interviews with eligible participants to collect information on socio-demographic factors and HL using C-HBP-HLS.

The C-HBP-HLS was developed by a three-phase process, as following.

#### Phase 1: Translation and development of the C-HBP-HLS

Firstly, the HBP-HLS was translated independently by two professional bilingual translators from English to Chinese. Secondly, the translation committee (two specialists in clinical nursing, two health management experts, three nursing educators, and one bilingual translator) checked and agreed on a version of the C-HBP-HLS that adapted to Chinese culture and accurately reflected the original English version of the HBP-HLS. Thirdly, two native bilingual translators with experience in medicine but unaware of the original English version of HBP-HLS completed a back-translation of the C-HBP-HLS from Chinese to English to establish semantic equivalence through comparison. After this, the final C-HBP-HLS version was achieved.

#### Phase 2: Content validity index (CVI)

The C-HBP-HLS was reviewed by seven experts (two hypertension specialists, two clinical nursing experts and three nursing educators). Each item was rated on a 4-point Likert scale ranging from 1 (strongly disagree) to 4 (strongly agree). The CVI of each item [item-level CVI (I-CVI)] was calculated to evaluate the C-HBP-HLS. The I-CVI ranged between 0.86 and 1.0 for each factor and the S-CVI/UA (universal agreement) of the C-HBP-HLS was 0.85, with all items retained. According to the suggestions of the experts, some of the items were revised to meet the cultural context in China. For example, Item 2.2 ‘‘And the next one after that?” was translated as ‘‘And when is the following third?”, Item 2.5 ‘‘Normal blood pressure is 120/80” was translated as ‘‘Ideal blood pressure is 120/80”, and Item 4.2 ‘‘If you are allowed to eat 2,400 milligrams of sodium per day, how many servings of ramen can you have?” was translated as ‘‘If you are allowed to eat 2,400 milligrams of sodium per day, how many bags of instant noodles can you have?”. A modified version of the C-HBP-HLS was subsequently developed.

#### Phase 3: Assessment of the psychometric properties

At first, the factor structure of the instrument was tested with EFA and CFA. Then, the internal consistency and test-retest reliability of the instrument were examined.

### Data analyses

Data were analyzed using SPSS18.0 and Amos18.0 software packages. Descriptive statistics were used to outline the demographic characteristics. The correlation between Q1, Q2, Q3 items and education level were computed using the correlation matrix. Mann-Whitney Test was used to compare the HL scores of the hypertension patients belonging to Kazakh and Han ethnicity in XinJiang. The reliability of C-HBP-HLS was assessed using internal consistency and test-retest reliability. Internal consistency was measured by Cronbach’s alpha coefficient. Construct validity was assessed by EFA and CFA. The EFA was conducted using Principal Axis Factoring Analysis with Equamax rotation to determine the underlying factor structure of the items. The factor retention was based on the following criteria: (a) eigenvalues greater than 1.0, (b) the percentage of total variance explained, (c) scree plot, and (d) factor loadings above 0.40,(e) item equal to or more than 2 being retained [24]. The CFA was performed using generalized least squares estimation to compare the current 5-factor model and the original 2-factor model of the scale, model fit was considered acceptable if χ^2^/df<2, adjusted goodness-of-fit index (AGFI)>0.9, comparative fit index(CFI)>0.9, goodness-of-fit index(GFI)>0.9, root mean square error of approximation(RMSEA)<0.06, and incremental fit index (IFI)>0.9 [25].

## Results

### Sample Characteristics

The demographic data regarding age, race, gender, education, blood pressure and years of hypertension were comparable between the pilot sample and the validation sample (*P*>0.05) (see Table 1).

**Table 1:** Demographic characteristics of the Study Participants (n = 550), 2013–2014.

Variables	Pilot Sample (n = 242)	Validation Sample (n = 308)
	Mean ±SD or n (%)	Mean ±SD or n (%)
Age (Range:17–88 years)	59.98±11.81	60.62±12.46
<40	17(7.0)	16(5.2)
40~59	76(31.4)	106(34.4)
≥60	149(61.6)	186(60.4)
Race		
Han Chinese	109(45.0)	136(44.2)
Kazakh Chinese	133(55.0)	172(55.8)
Gender		
Male	118(48.8)	151(49.0)
Female	124(51.2)	157(51.0)
Education		
Less than high school	131(54.1)	166 (53.9)
High school or higher	111(45.9)	142(46.1)
Blood pressure (BP, mmHg)		
Systolic BP	148.63±22.42	149.61±22.75
Diastolic BP	82.67±14.56	83.02±15.04
Years of hypertension (years)		
≤5	126(52.1)	149(48.4)
>5	116(47.7)	159(51.6)

### Construct Validity

**Fig 1:**
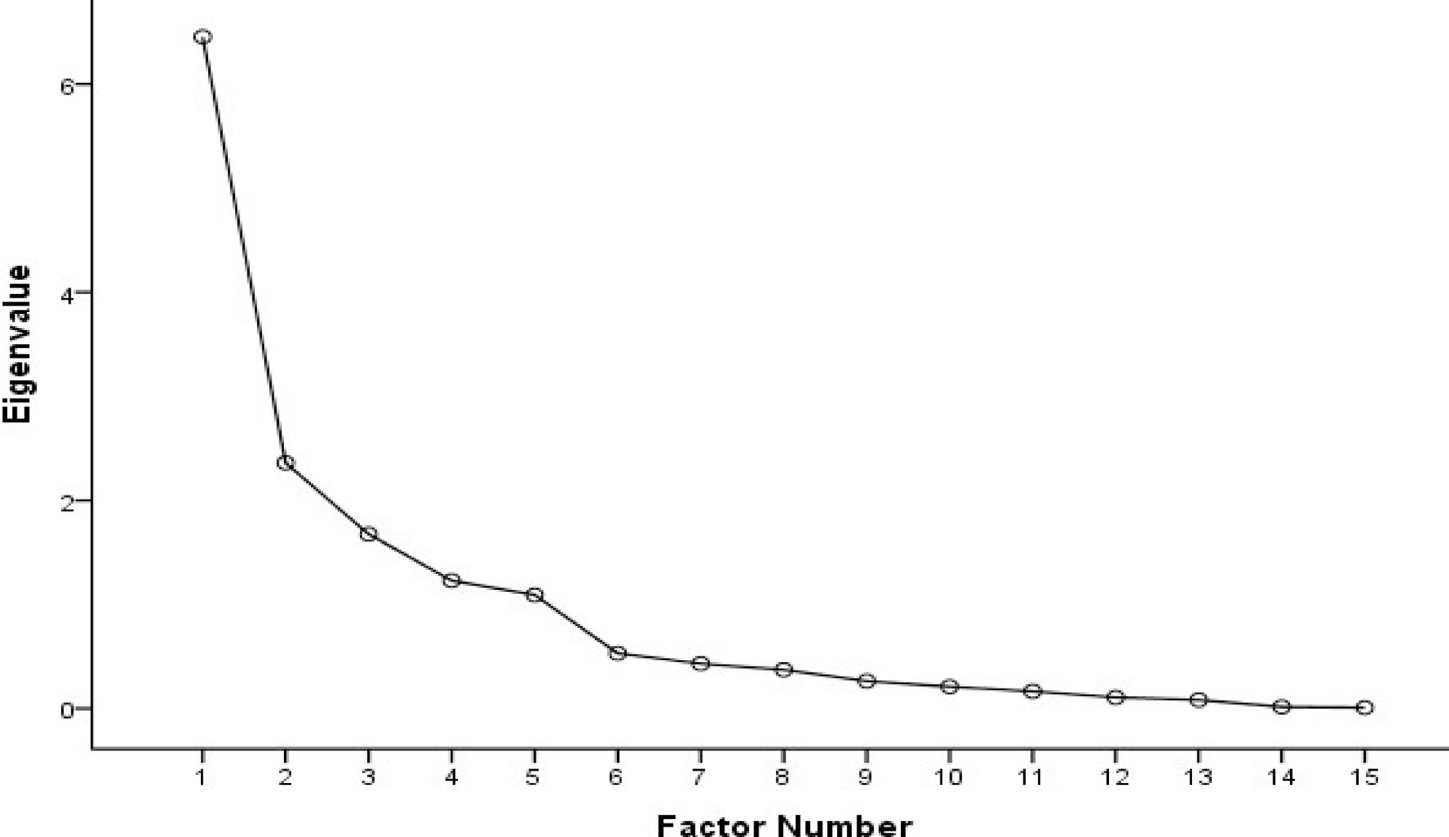
Scree Plot of the C-HBP-HLS.

Bartlett’s test of sphericity was found to be significant (χ^2^ = 4046, *P*<0.001). The Kaiser-Meyer-Olkin (KMO) value was 0.824. During EFA, five factors were extracted and the item 2.5 was excluded as the factor loading was lower than 0.4. The items 2.3, 2.4, 3.1 and 3.2 together formed a new dimension. According to the contents of items and the results of the Scree plot (Fig 1), the C-HBP-HLS retained 15 items and displayed 5-factor structure, which explained 77.7% of the total sample variance. As shown in Table 2, Factor 1 (3 items), was consistent with all items of the original scale’s print literacy, which also retained the original name “Print HL”. Factor 2 (2 items), Factor 3 (4 items), Factor 4 (4 items), and Factor 5 (2 items), were respectively termed as “Medication Label”, “Understanding Ability”, “Newest Vital Sign Test” and “Avoiding food allergy”. “Medication Label” referred to the correct medication interval time. “Understanding Ability” meant that the participants could understand instructional messages and appointment slip content. “Newest Vital Sign Test” encompassed dietary approach to stop hypertension using a bag of instant noodles, and “Avoiding food allergy” referred to the understanding and prevention of food allergy. All these 4 factors did constitute the Functional HL-dimension of the original HBP-HLS.

**Table 2:** Results of Exploratory Factor Analysis (n = 242), 2013–2014.

Item	Factors				
	1	2	3	4	5
1.1 Print literacy1	0.913				
1.2 Print literacy2	0.917				
1.3 Print literacy3	0.909				
2.1 If you take your first tablet at 7:00 am, when should you take the next one		0.894			
2.2 And when is the following third?		0.972			
2.3 If you have a lunch at noon and plan to take the medication **BEFORE a meal**, what time do you have to take the medication?			0.610		
2.4 If you have a lunch at noon and plan to take the medication **AFTER a meal,** what time do you have to take the medication?			0.847		
3.1 When is the appointment date?			0.520		
3.2 Where?			0.537		
4.1 If you eat the entire bag, how many calories will you eat?				0.819	
4.2 If you are allowed to eat 2,400 milligrams of sodium per day, how many bags of instant noodles can you have?				0.798	
4.3Your doctor advises you to reduce the amount of saturated fat in your diet. If you decide not to eat a bag of Ramen today, how many grams of saturated fat would you be reducing?				0.825	
4.4 If you usually eat 2900 calories a day, what percentage of your daily value of calories will you be eating if you eat one serving of this Ramen?				0.688	
4.5 Is it safe for you to eat this Ramen?					0.809
4.6 (Ask only if the patient responds “no” to question 4.5): Why not?					0.745
Eigenvalues	3.547	2.903	2.045	1.852	1.306
% of variance	23.648	19.3521	13.636	12.347	8.706
Cumulative%	23.648	43.000	56.636	68.983	77.688

**Fig 2:**
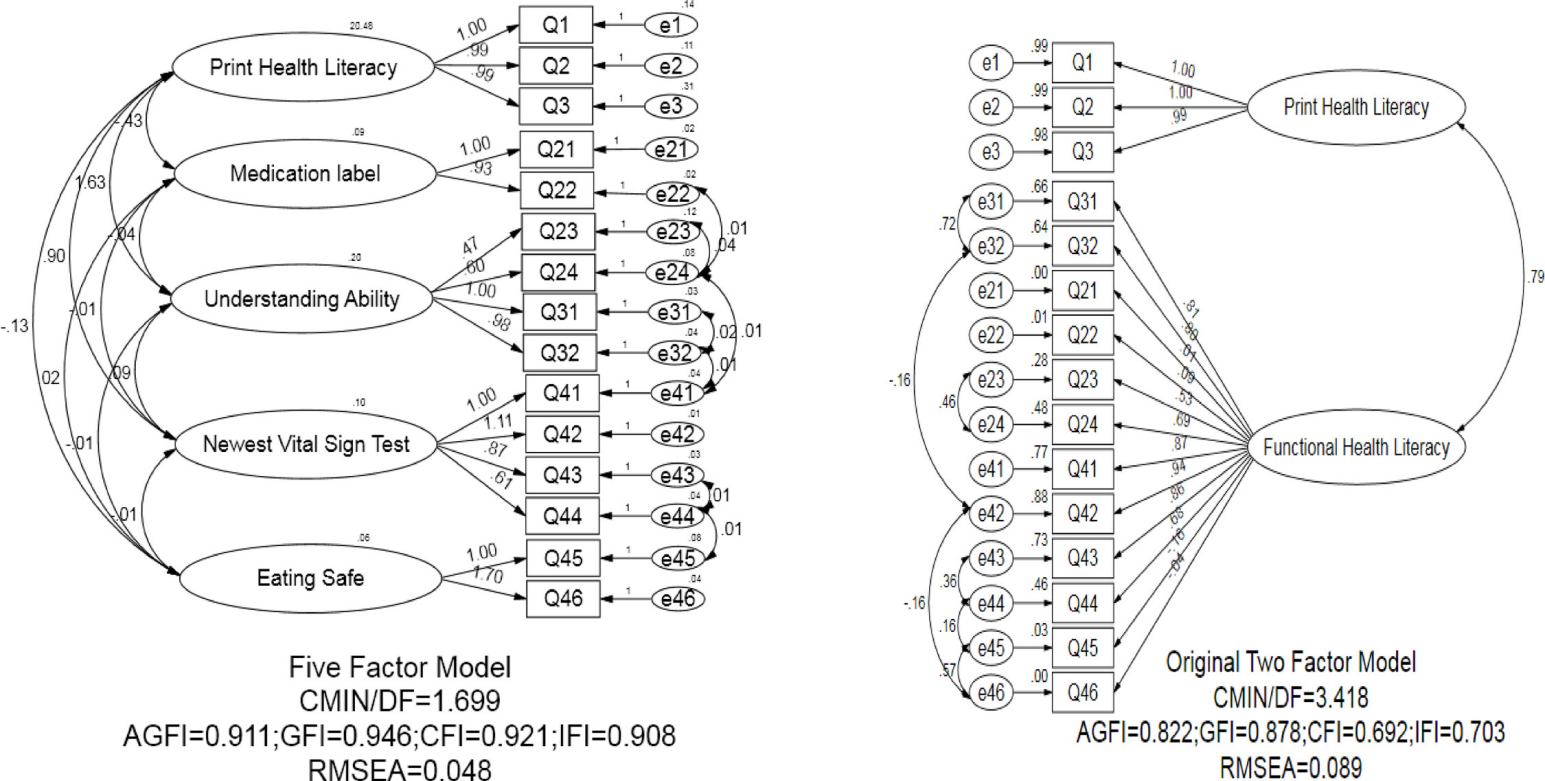
The five-and two-factor model of the HBP-HLS with standardized estimates fitted in a sample of Chinese hypertension patients (n = 308), 2013–2014

CFA was used to establish the most appropriate factor structure of the C-HBP-HLS, the 5-factor model was found to fit the data much better with a smaller model χ^2^ statistic (χ^2^ = 124.032, *P* = 0.000) and stronger model fit indexes (AGFI = 0.91, GFI = 0.95, RMSEA = 0.048, and IFI = 0.92), than the original 2-factor model (χ^2^ = 280.262, *P* = 0.000, AGFI = 0.82, GFI = 0.88, RMSEA = 0.10, and IFI = 0.70) [17] (Fig 2). Because CFA was sensitive to sample size, the Chi-square value was significant (*P*<0.05), but this did not necessarily indicate poor fit [26]. Differences in Chi-square values (△χ^2^) across models might be more informative than the chi-square values themselves. So, in this study, the △χ^2^ and relative fit indexes were used to estimate how well model explained the data [27]. The original 2-factor model χ^2^ –value was less satisfactory than the 5-factor model (△χ^2^ = 156.23). All standardized factor loadings were greater than 0.5 in the 5-factor model. As expected, the C-HBP-HLS with 5-factor structure model was an acceptable fit of the model.

Given the significant differences between Han-Chinese and Kazakh-Chinese, we also separated Han-Chinese and Kazakh-Chinese for Exploratory Factor Analysis (S1A and S1B Appendix).

### Reliability Testing

The Cronbach’s alpha coefficient was 0.78 for the total scale and ranged between 0.72 and 0.99 for each factor. The total test-retest reliability coefficient over two week’s interval for the subsample of 37 participants was 0.96, and ranged between 0.87 and 0.96 for each factor (see Table 3).

**Table 3:** Internal Consistency and Test-Retest reliability of the C-HBP-HLS, 2013–2014.

	Items	Mean ±SD (n = 242)	Range	Cronbach’s α (n = 242)	Test-retest reliability(n = 37)
C-HBP-HLS (Print HL+ Functional HL)	15	18.31±15.96	0–42	0.779	0.960^*^
Print HL	3	12.31±14.15	0–30	0.995	0.950^*^
Functional HL	12	6.0±2.92	0–12	0.810	0.933^*^
Medication Label	2	1.76±0.63	0–2	0.938	0.959^*^
Understanding Label	4	1.67±1.61	0–4	0.837	0.916^*^
Newest Vital Sign Test	4	0.95±1.50	0–4	0.907	0.871^*^
Avoiding Food Allergy	2	1.62±0.68	0–2	0.719	0.950^*^

^*^P <0.01

#### All participants’ HL score

Considering the item Q1, Q2, Q3 factor loadings were greater than 0.95, we tested the correlation between Q1, Q2, Q3 items and education level within the pilot sample as well as the validation sample (n = 550). Education level was found to have a strong positive correlation with the scores determined by the Q1, Q2, and Q3 items (r = 0.481, 0.492, 0.475, respectively, *P*<0.01). It was also observed that the HL score of the subjects belonging to Kazakh population in the study area was significantly lower than that among the Han community (7.13±7.90 & 30.10±13.42, respectively, Z = -14.573, *P*<0.001, Table 4).

**Table 4:** Health literacy scores of the C-HBP-HLS between Kazakh and Han Chinese (n = 550), 2013–2014.

Populations	n	Total score of C-HBP-HLS	Print HL	Functional HL
Kazakh-Chinese	305	7.13±7.90	2.44±6.84	4.69±1.87
Han-Chinese	245	30.10±13.42	23.47±11.33	6.63±3.43
Z		-14.573	-17.016	-6.651
*p*		<0.001	<0.001	<0.001

## Discussion

The main purpose of this study was to translate and validate HBP-HLS in a community-based sample of hypertensive patients belonging to Kazakh ethnic minority population and Han community in Urumqi, XinJiang, western China. Till the initiation of this study, quite a few HL-measurement instruments including the 14-item HL Scale (HLS-14) [28] and Adult Literacy in Dentistry (AREALD-30) [29] were developed and evaluated by cross-validation of EFA and CFA. However, to the best of our knowledge, in Chinese context, this was the first effort to develop a hypertension-specific HL measurement tool, validate it by combining the methods of EFA and CFA and to test its psychometric properties. The translation process was conducted rigorously to ensure the equivalence between scales, and the findings of the psychometric analyses of C-HBP-HLS were promising.

For the proposed validation, use of an independent sample to confirm the hypothesized model and test the structure of the theory were required, instead of trying to confirm the initial model using the same sample as the later method was likely to boil down to a mere data fitting [27]. It was thus recommended that the validation of EFA and CFA should be tried using two different samples. Consequently, in our study, an independent random sample was used to evaluate the Goodness-of-Fit of the proposed model. Findings of the study indicated that the factors (five) extracted from the data through EFA, were adequate to capture the important features of hypertension-specific HL and variations thereof, as the cumulative contribution of these factors was found to be 77.7%, In Chinese cultural context, dimension Functional HL of the original scale was further divided into four sub-dimensions, namely Medication Label, Understanding Ability, Newest Vital Sign Test and Avoiding Food Allergy. In this study, the factorial structure of the C-HBP-HLS was evaluated by comparing two different factor solutions (2 and 5 factors model) in an independent adult population of hypertensive patients in China. CFA revealed that 5-factor model provided a Goodness-of-fit with the data, but the original 2-factor model had an unacceptable fit (△χ^2^ = 156.23). Thus it appeared that compared to the original 2-factor model, the 5-factor model was more suitable and sensitive to capture the main features of HL among Chinese hypertensive patients.

In addition to the explained difference between the initial and the finalized tool, modeled through factor analyses, numbers of items were also minimized (from 16 in the initial to 15 in the final one) based on the results. The item no. 2.5 “Your blood pressure today is 140/100. Is this normal?” was deleted, due to the factor load being <0.4. Probable explanation included the possibilities that this item could not belong to any of the dimensions of the scale, or the patients might have mostly guessed the answer to this question based on their own experiences not according to the prompts provided. What's more, the item 2.2“If you have a lunch at noon and plan to take the medication BEFORE a meal, what time do you have to take the medication?”, the item 2.3“If you have a lunch at noon and plan to take the medication AFTER a meal, what time do you have to take the medication?”, the item 3.1“When is the appointment date?”, and the item 3.2 “Where is the appointment location?” constituted a new dimension named as " Understanding Ability ". The findings suggested that individuals who could answer these questions were able to show transition to higher levels of cognition, such as analyzing, understanding, and applying instructional messages and appointment slip. The factor Loading for the Q1, Q2 and Q3 item (reading 30 hypertension-related words arranged in 3 groups) was more than 0.95. The findings could be explained by the observed strong positive correlation between the education level of the subjects and the scores for these 3items indicating that the higher was the education level of the patient, the better reading skills s/he had.

The current study showed that the S-CVI /UA of C-HBP-HLS was 0.85, suggesting that C-HBP-HLS could clearly meet the requirement (>0.8) and authenticity regarding what it was supposed to measure [20]. Moreover, C-HBP-HLS had a satisfactory internal consistency, with a Cronbach's alpha of 0.78 [30]. It did also reveal a high retest reliability during a 2-week period, with test-retest reliability coefficient across all domains varying between 0.87 and 0.96. These results cumulatively emphasized satisfactory reliability and adequate robustness of the developed instrument.

The findings of the psychometric analyses suggested that the C-HBP-HLS was a reliable and valid tool for the assessment of HL among hypertensive patients in China, and had the potential for being considered as an instrument to increase the effectiveness of the hypertension-control programs in this country by providing accurate and comprehensive advice to the patients for controlling blood pressure. As a significant independent predictor of the effectiveness of blood pressure control programs [13], assessment of HL among hypertensive patients could provide essential information regarding their level of HL and associated factors, and as well as for further development of relevant interventions. Thus, the requirement of further research for the identification of the correlates of HL along with measurement of the strength of association between HL and other indexes (e.g. self-management) among hypertension patients were clearly evident from the finding of this study. Due to the space constraints, the detailed patterns of the responses to C- HBP-HLS provided by the patients’ will be presented elsewhere.

### Limitation of the study

It is important to note that in this study, hypertensive patients belonging to Han and Chinese Kazakh communities were recruited from the same city, which might have limited the generalizability of the findings of this study. Thus any effort to use this tool among other Chinese-speaking ethnic population elsewhere might need appropriate validation of the questionnaire to relevant communities and geographical regions. Another major limitation of our study was the issue that, owing to the lack of "golden criterion" for the measurement of hypertension-specific HL, criterion validity could not be measured.

## Conclusions

The validated version of C-HBP-HLS consisted of five dimensions with 15 items, including Print HL, Medication Label, Understanding Ability, Newest Vital Sign Test, and Avoiding Food Allergy. This tool was found to be a reliable and valid one for the assessment of HL among Hypertension patients in China. As a robust instrument with excellent psychometric properties, C-HBP-HLS appeared to have the potential for providing adequate insight to the health professionals to design efficient and appropriately focused interventions targeting the improvement of HL and self-management among hypertensive patients in China.

## Supporting Information

S1 Appendix Results of Exploratory Factor Analysis among Han Chinese (n = 245), 2013–2014 and results of Exploratory Factor Analysis among Kazakh Chinese (n = 305), 2013–2014. (DOC)
