# Supplementary material for: Cross-Cultural Validation of the High Blood Pressure Health Literacy Scale in a Chinese Community
Source: PLoS One. 2016 Apr 26;11(4):e0152182. doi: 10.1371/journal.pone.0152182 (PMC4846086; doi:10.1371/journal.pone.0152182)
Supplement: S1 Appendix — (DOC) [file pone.0152182.s001.doc]

**Appendix**

**Appendix A. Results of Exploratory Factor Analysis among Han Chinese (n= 245), 2013-2014**

| Item | Factors | | | |  |
| --- | --- | --- | --- | --- | --- |
|  | 1 | 2 | 3 | 4 | 5 |
| 1.1 Print literacy1 | 0.934 |  |  |  |  |
| 1.2 Print literacy2 | 0.945 |  |  |  |  |
| 1.3 Print literacy3 | 0.933 |  |  |  |  |
| 2.1 If you take your first tablet at 7:00 am, when should you take the next one |  | 0.896 |  |  |  |
| 2.2 And the next one after that?  2.3 If you have a lunch at noon and plan to take the medication **BEFORE a meal**, what time do you have to take the medication?  2.4 If you have a lunch at noon and plan to take the medication **AFTER a meal,** what time do you have to take the medication? |  | 0.952 | 0.589  0.666 |  |  |
| 3.1 When is the appointment date? |  |  | 0.766 |  |  |
| 3.2 Where? |  |  | 0.764 |  |  |
| 4.1 If you eat the entire bag, how many calories will you eat? |  |  |  | 0.802 |  |
| 4.2 If you are allowed to eat 2,400 milligrams of sodium per day, how many servings of ramen can you have? |  |  |  | 0.812 |  |
| 4.3Your doctor advises you to reduce the amount of saturated fat in your diet. If you decide not to eat a bag of Ramen today, how many grams of saturated fat would you be reducing? |  |  |  | 0.853 |  |
| 4.4 If you usually eat 2900 calories a day, what percentage of your daily value of calories will you be eating if you eat one serving of this Ramen? |  |  |  | 0.647 |  |
| 4.5 Is it safe for you to eat this Ramen? |  |  |  |  | 0.892 |
| 4.6 (Ask only if the patient responds “no” to question 4.5): Why not? |  |  |  |  | 0.580 |
| Eigenvalues | 3.053 | 2.759 | 2.371 | 2.324 | 1.278 |
| % of variance | 20.355 | 18.394 | 15.810 | 15.492 | 8.517 |
| Cumulative% | 20.355 | 38.749 | 54.559 | 70.050 | 78.567 |

**Appendix B Results of Exploratory Factor Analysis among Kazakh Chinese (n= 305), 2013-2014**

| Item | Factors | | | |  |
| --- | --- | --- | --- | --- | --- |
|  | 1 | 2 | 3 | 4 | 5 |
| 1.1 Print literacy1 | 0.952 |  |  |  |  |
| 1.2 Print literacy2 | 0.959 |  |  |  |  |
| 1.3 Print literacy3 | 0.941 |  |  |  |  |
| 2.1 If you take your first tablet at 7:00 am, when should you take the next one |  | 0.859 |  |  |  |
| 2.2 And the next one after that?  2.3 If you have a lunch at noon and plan to take the medication **BEFORE a meal**, what time do you have to take the medication?  2.4 If you have a lunch at noon and plan to take the medication **AFTER a meal,** what time do you have to take the medication? |  | 0.873 | 0.674  0.789 |  |  |
| 3.1 When is the appointment date? |  |  | 0.696 |  |  |
| 3.2 Where? |  |  | 0.700 |  |  |
| 4.1 If you eat the entire bag, how many calories will you eat? |  |  |  | 0.796 |  |
| 4.2 If you are allowed to eat 2,400 milligrams of sodium per day, how many servings of ramen can you have? |  |  |  | 0.849 |  |
| 4.3Your doctor advises you to reduce the amount of saturated fat in your diet. If you decide not to eat a bag of Ramen today, how many grams of saturated fat would you be reducing? |  |  |  | 0.891 |  |
| 4.4 If you usually eat 2900 calories a day, what percentage of your daily value of calories will you be eating if you eat one serving of this Ramen? |  |  |  | 0.880 |  |
| 4.5 Is it safe for you to eat this Ramen? |  |  |  |  | 0.924 |
| 4.6 (Ask only if the patient responds “no” to question 4.5): Why not? |  |  |  |  | 0.912 |
| Eigenvalues | 3.196 | 3.035 | 2.236 | 1.743 | 1.513 |
| % of variance | 21.304 | 20.235 | 14.904 | 11.622 | 10.087 |
| Cumulative% | 21.304 | 41.539 | 56.443 | 68.065 | 78.152 |
